# Long-term outcomes of lamellar crescentic wedge resection on corneal astigmatism and optical aberrations in patients with advanced pellucid marginal degeneration

**DOI:** 10.55730/1300-0144.6199

**Published:** 2026-02-15

**Authors:** Ayşe BURCU, Hande Hüsniye TELEK, Hakan MERT, Yaprak Arzu ÖZDEMİR, Züleyha Yalnız AKKAYA, Evin ŞİNGAR, Selma Özbek UZMAN

**Affiliations:** 1 Department of Ophthalmology, Ankara Training and Research Hospital, University of Health Sciences, Ankara, Turkiye; 2 Department of Statistics, Faculty of Science, Gazi University, Ankara, Turkiye

**Keywords:** Pellucid marginal degeneration, lamellar wedge resection, corneal astigmatism, corneal aberrations

## Abstract

**Background/aim:**

The present study investigates the long-term changes in corneal astigmatism and aberrations following lamellar wedge resection in cases with advanced pellucid marginal degeneration (PMD).

**Materials and methods:**

Patients with advanced PMD whose visual acuity did not improve with glasses or contact lenses underwent lamellar crescentic wedge resection. Pre- and postoperative assessments at 3, 6, 12, and 24 months using Pentacam HR were conducted to identify changes in corrected visual acuity, corneal astigmatism, and higher-order aberrations (HOAs) following the procedure, and compared with a control group.

**Results:**

Statistically significant postoperative improvements in corrected visual acuity were observed at 6 and 12 months compared to preoperative values. At postoperative 3, 6, 12, and 24 months, significant improvements from preoperative values were noted in logMAR, astigmatism, and Kmax parameters (p < 0.05). A comparison of the patients with PMD and a control group revealed significant postoperative reductions in anterior and posterior coma, trefoil, spherical, and tetrafoil aberrations at 4 mm and 6 mm (p < 0.05). Most parameters remained statistically different from the healthy controls, indicating improvement without complete normalization. Although no significant difference was noted in the 6 mm anterior root mean square (RMS) value, a statistically significant reduction was observed in the 6 mm posterior RMS value at postoperative 3 and 6 months (p < 0.05).

**Conclusion:**

Lamellar crescentic wedge resection may be considered an appropriate and relatively safe surgical option for selected patients with advanced PMD, avoiding the risks associated with open-eye surgery, graft rejection, and secondary glaucoma. Long-term improvements in visual acuity, corneal astigmatism, and selected HOAs were noted following the procedure. Given the limited sample size and exploratory study design, these findings should be interpreted with caution and considered hypothesis-generating rather than definitive.

## 1. Introduction

Pellucid marginal degeneration (PMD) is an uncommon, noninflammatory corneal ectatic disorder that typically presents as a slowly progressive condition in individuals over 30 years of age. The disease is characterized by crescent-shaped inferior corneal thinning, usually located 1–2 mm from the inferior limbus. This area of thinning typically extends between the 4 o’clock and 8 o’clock meridians and is approximately 1–2 mm in width. PMD leads to progressive irregular astigmatism and visual deterioration and is commonly recognized by the characteristic “butterfly” or “crab claw” pattern observed on corneal topography and tomography [1–3].

Early-stage PMD is primarily managed conservatively and includes optical correction with spectacles or various types of contact lenses, such as soft lenses, rigid gas-permeable lenses, hybrid lenses, piggyback systems, and scleral lenses. However, in patients who experience significant intolerance to contact lenses or demonstrate disease progression with inadequate visual rehabilitation, surgical intervention may become necessary. Surgical options include intracorneal ring segment implantation, corneal wedge resection (lamellar or full-thickness), deep anterior lamellar keratoplasty, and penetrating keratoplasty. Recent studies suggest that corneal collagen cross-linking may play a role in stabilizing disease progression in selected PMD cases [4–6].

Lamellar wedge resection has emerged as an alternative surgical approach to the correction of advanced and highly irregular astigmatism, given the limited effectiveness of intracorneal ring segments and the potential complications associated with full-thickness wedge resection and penetrating keratoplasty—such as secondary glaucoma, surgically induced irregular astigmatism, and risks related to open-sky procedures. The technique offers several potential advantages, including preservation of central corneal tissue, enhanced wound stability, faster visual rehabilitation, reduced exposure to postoperative corticosteroids, and a lower risk of immunological rejection compared to full-thickness procedures [7–10].

More recently, wavefront-based analysis has been increasingly used to evaluate corneal optical quality by quantifying higher-order aberrations (HOAs), which are commonly described using Zernike polynomial decomposition. While wavefront technology provides a more comprehensive assessment of corneal irregularity beyond conventional keratometric measurements, knowledge of long-term changes in corneal HOAs following surgical intervention in PMD remains limited [11–14]. The present study investigates the long-term changes in corneal astigmatism and selected HOAs following lamellar wedge resection in patients with advanced PMD.

## 2. Materials and method

Included in this retrospective case-control study were single eyes of seven patients who underwent lamellar crescentic wedge for advanced PMD. All patients had experienced insufficient visual improvement with spectacles or contact lenses prior to surgery and were followed-up regularly for up to 24 months postoperatively. A control group was included for comparative analysis, consisting of eight age-matched individuals with best-corrected visual acuity of 20/20 or better, no history of ocular disease or surgery, and no systemic conditions known to affect the cornea. All control eyes underwent comprehensive ophthalmological examination and Scheimpflug-based corneal tomography, and only those demonstrating normal anterior and posterior corneal curvature, thickness distribution, and topographic indices, with no evidence of corneal ectasia or irregularity, were included in the study.

The exclusion criteria for both groups included a history of ocular surgery or trauma, presence of keratoconus or other corneal ectatic disorders, corneal dystrophies, ocular allergy, or any other corneal pathology, a history of autoimmune or collagen vascular disease, use of systemic or topical medications affecting the eye, and systemic conditions such as diabetes mellitus.

Informed consent was obtained from all participants prior to their inclusion in the study. The study was conducted in accordance with the principles of the Declaration of Helsinki, and ethical approval was obtained from the Local Ethics Committee on December 2023 (approval ID: E-23-1469).

All eyes underwent standardized preoperative and postoperative ophthalmic examinations, including uncorrected visual acuity, autorefraction, intraocular pressure measurement, slit-lamp biomicroscopy, and Scheimpflug-based corneal tomography. Corneal imaging was performed using the Pentacam HR system (Oculus Optikgeräte GmbH, Wetzlar, Germany).

### 2.1. Surgical technique

All surgical procedures were performed under general anesthesia using standard sterile techniques. After strict aseptic and antiseptic preparation, the planned excision area was carefully marked under an operating microscope based on the location and extent of inferior corneal thinning.

A crescent-shaped blade was used to excise the ectatic corneal tissue, typically measuring approximately 8–10 mm in arc length and 2–4 mm in width, depending on the severity and extent of corneal thinning. The wedge-shaped resection included the corneal epithelium, subepithelial tissue, and anterior stromal layers, while the posterior stroma and Descemet membrane were preserved.

Following lamellar wedge excision, a 1.2-mm side-port incision was created to temporarily reduce intraocular pressure and facilitate wound apposition. The resection site was then closed with interrupted 10-0 monofilament nylon sutures. All surgical procedures were performed by a single experienced surgeon (AB), ensuring procedural consistency.

Postoperative management included topical moxifloxacin eye drops administered eight times daily for 1 week, topical dexamethasone eye drops applied eight times daily and gradually tapered over a 3-month period, and preservative-free artificial tears used four times a day.

### 2.2. Statistical analysis

Statistical analyses were performed using IBM SPSS Statistics, Version 22.0 (IBM Corp., 2011). Data distribution was assessed using the Shapiro–Wilk test. As at least one of the variables included in the analyses did not meet the normality assumption, nonparametric statistical methods were used.

To evaluate changes in repeated measurements prior to surgery and at postoperative months 3, 6, 12, and 24 within the PMD group, the Friedman test was used. When statistically significant differences were detected, post hoc pairwise comparisons were performed using Dunn’s test. p-values were adjusted using the Bonferroni correction for multiple comparisons to control for Type I error. Statistical significance was set at an adjusted p-value of <0.05. Descriptive statistics were reported as mean ± standard deviation, along with minimum and maximum values to provide a clear overview of the data characteristics.

Differences between the PMD and control groups were analyzed using the Mann–Whitney U test, as the data violated the assumption of normality. This nonparametric approach was chosen to maintain adequate statistical power given the limited sample size. Results were considered statistically significant at p < 0.05.

## 3. Results

The patient group (n = 7) comprised four males (62.5%) and three females (37.5%), with a mean age of 51.37 ± 15.57 years, and was matched with eight individuals in the control group of four males (50%) and four females (50%), with a mean age of 55.12 ± 18.35 years. No statistically significant differences were observed between the patient and control groups in terms of age or sex (p >0.05).

The evaluated parameters included best-corrected visual acuity (logMAR), astigmatism, axis, Kmax, and HOAs. HOA analysis included the evaluation of the individual aberration components—namely the anterior and posterior coma, spherical aberration, trefoil, and tetrafoil—as well as total higher-order aberrations, expressed as root mean square (RMS). Measurements were obtained from the 4 mm and 6 mm optical zones, preoperatively and at postoperative months 3, 6, 12, and 24. Aberration analyses were carried out using the 4 mm and 6 mm analysis zones using the Scheimpflug-based topography system. The 4 mm zone primarily represents the central optical area under photopic conditions, whereas the 6 mm zone reflects optical performance under mesopic and scotopic conditions, when pupil dilation increases the contribution of peripheral corneal irregularities. Accordingly, postoperative changes were analyzed both longitudinally within the PMD group and comparatively against the control group, allowing the assessment of localized aberration behavior as well as cumulative optical distortion.

A comparison of the parameters among the PMD patients prior to surgery and at postoperative months 3, 6, 12, and 24 is presented in Table 1. Friedman’s test identified statistically significant differences in repeated measures for logMAR visual acuity, 4 mm anterior trefoil, and 4 mm posterior coma (p < 0.05), while parameters without significant temporal variation were excluded from the table. Post-hoc analysis revealed a significant improvement in logMAR values between the preoperative measurements and those obtained at postoperative months 6 (p = 0.014) and 12 (p = 0.014).

Friedman’s test also revealed a statistically significant change over time in the 4 mm anterior trefoil parameter (p < 0.05). Post hoc analysis showed a significant reduction in 4 mm anterior trefoil values between the preoperative and 6-month postoperative measurements (p = 0.039). Further significant decreases were noted between postoperative months 3 and 24 (p = 0.020), and between postoperative months 6 and 24 (p = 0.002), with the lowest values recorded at 24 months. These findings indicate a progressive and sustained reduction in this dominant aberration component within the central optical zone following lamellar wedge resection. The sustained reduction within the 4 mm optical zone indicates improvement in central optical quality, which is particularly relevant for visual performance under normal lighting conditions. Moreover, post hoc analysis of 4 mm posterior coma revealed a significant reduction between the preoperative and 3-month postoperative values (p = 0.027).

The PMD patient and control group measurements were compared statistically according to data distribution, and the findings related to logMAR visual acuity, astigmatism, axis, and Kmax are summarized in Table 2. Significant differences were observed between the two groups in logMAR, astigmatism, and Kmax values at all evaluated time points (p < 0.05), while no statistically significant differences were observed in axis values between the two groups.

Analysis of 4 mm HOAs (Table 3) revealed significantly higher anterior coma, trefoil/tetrafoil, and spherical aberration values in the PMD patient group compared with the controls, both preoperatively and at all postoperative time points (p <0.05). Although postoperative reductions were observed in the individual aberrations, the differences remained significant when compared to the control group. Notably, no statistically significant differences were noted between the anterior 4 mm RMS values of the PMD patients and the control group at any preoperative or postoperative time point (p > 0.05). This suggests that optical degradation within the central 4 mm optical zone in PMD is primarily driven by selective increases in specific aberration components—particularly coma, trefoil, and tetrafoil—rather than by a uniform increase in total higher-order aberration.

Posterior 4 mm HOA analysis revealed significantly higher posterior coma values in the PMD patients at the preoperative stage and at the 12- and 24-month postoperative time points compared with the control group (p < 0.05). Posterior trefoil/tetrafoil and spherical aberration values were also significantly elevated preoperatively and at selected postoperative time points; however, the posterior 4 mm RMS values did not differ significantly between the PMD patients and the control group at any time point (p > 0.05), reinforcing the finding that aberration changes within the smaller optical zone are predominantly component-specific rather than cumulative.

Our evaluation of 6 mm HOAs (Table 4) revealed more pronounced and persistent differences between the PMD patients and the control group. Notably, significant differences were observed in the anterior 6 mm coma, trefoil, and tetrafoil values at the preoperative stage and at several postoperative time points (p < 0.05). The anterior spherical aberration values also differed significantly between the groups at selected intervals, indicating that aberrations affecting the larger optical zone remain prominent even after surgery. Furthermore, aberrations affecting the larger optical zone may be more relevant under pupil dilation, such as under dim illumination or nighttime viewing conditions.

While no statistically significant differences were observed between the anterior 6 mm RMS values of the two groups, the posterior 6 mm RMS values demonstrated significant differences at the 3- and 6-month postoperative time points (p < 0.05). This increase in total posterior higher-order aberration within the 6 mm zone suggests greater cumulative optical distortion under mesopic or scotopic conditions, which may be associated with symptoms such as glare, halos, and reduced night-vision quality.

Serial anterior segment images of one of our patients with PMD who underwent lamellar wedge are presented in Figure 1: Preoperatively (Figure 1a), postoperative month 3 (Figure 1b), postoperative month 6 (Figure 1c), and postoperative month 12 (Figure 1d).

Pentacam images of the same patient are presented in Figure 2: Preoperatively (Figure 2a) and at postoperative month 20 (Figure 2b).

## 4. Discussion

A variety of surgical techniques can be applied for the management of PMD, reflecting the heterogeneous presentation and progressive nature of the disease [15–17]. In advanced stages, surgical interventions such as penetrating keratoplasty and deep anterior lamellar keratoplasty are commonly employed to reduce high corneal astigmatism and improve visual acuity [18–20]. However, visual outcomes following these procedures in PMD are often less predictable than those reported for keratoconus, largely due to the peripheral and inferior localization of the ectatic area, which limits the ability to achieve optimal optical regularization [21–23].

To stabilize disease progression, corneal collagen cross-linking has been widely adopted in selected cases [24–26]. More recently, novel combined approaches, including sliding keratoplasty integrated with transepithelial iontophoresis-assisted collagen cross-linking, have been introduced in an effort to improve biomechanical stability while minimizing surgical invasiveness [27].

The concept of corneal wedge resection for PMD was first introduced by Duran et al. in 1991 [28]. In recent decades, lamellar wedge resection has emerged as a favorable alternative to full-thickness procedures, offering such advantages as preservation of the central cornea, improved wound stability, faster visual rehabilitation, reduced need for prolonged corticosteroid use, and a lower risk of immunological rejection [8,29,30].

The technique involves the selective excision of ectatic peripheral corneal tissue and the creation of a more physiologic corneal contour. Unlike full-thickness wedge resections, which inherently involve open-eye surgery and carry a greater risk of intraoperative and postoperative complications, lamellar wedge resection allows for dissection as close as possible to Descemet’s membrane while maintaining globe integrity. The approach facilitates the creation of a smoother corneal surface, thereby reducing the risk of interface haze and postoperative irregular astigmatism, both of which are critical determinants of visual quality in PMD patients.

Despite these advantages, previous studies of lamellar wedge resection in PMD remain limited, primarily due to the rarity of the disease itself [31–33]. Busin et al. reported the outcomes of lamellar wedge resection combined with penetrating relaxing incisions in 10 eyes with PMD, demonstrating significant improvements in corrected visual acuity and corneal astigmatism in eight patients at 6 months follow-up [34]. Similarly, Maccheron et al. reported improved visual acuity and a marked reduction in astigmatism in seven of 10 PMD patients evaluated at postoperative month 10 [35].

MacLean et al. evaluated full-thickness crescentic wedge resection in 10 eyes with PMD and reported postoperative improvement in best-corrected visual acuity (BCVA) in all cases, along with a substantial reduction in keratometric astigmatism from a mean of 13.8 D to 1.4 D [8]. However, long-term follow-up revealed a progressive astigmatic shift averaging 2.1 D, with individual variations ranging from 0.5 to 5.5 D. In addition, suture-related complications, including inferior pannus formation in three patients and corneal hydrops in one patient, were reported.

In contrast, Genç et al. found no evidence of astigmatic regression, corneal vascularization, or suture-related complications during a mean follow-up period of 14 months following lamellar wedge resection [12]. Kymionis et al. combined wedge resection with collagen cross-linking, and reported significant early postoperative improvements in visual acuity and corneal astigmatism; however, irregularities in corneal topography had become evident by the second postoperative month, highlighting the complex biomechanical response of the cornea following combined interventions [36].

Given the rarity of PMD, the relatively small sample sizes reported in these studies are consistent with the existing literature. Notably, previous reports have tended to focus primarily on visual acuity and keratometric outcomes, while evaluations of HOAs and short- to midterm follow-up periods are limited. In this regard, the present study is distinctive in its long-term patient follow-up of up to 24 months and its comprehensive analysis of both individual HOAs and total aberration metrics, offering a more detailed understanding of the optical quality changes following lamellar wedge resection in PMD.

In the present study, a significant improvement in corrected visual acuity was observed from baseline in patients with PMD at both the 6- and 12-month postoperative time points following lamellar wedge resection. In addition, when compared with the healthy control group, statistically significant differences in logMAR visual acuity, corneal astigmatism, and Kmax values were observed at postoperative months 3, 6, 12, and 24, whereas axis values did not differ significantly between the two groups. These findings suggest that lamellar wedge resection effectively improves visual performance and corneal steepness parameters, although complete normalization of corneal optics may not be achieved in all cases.

Studies of corneal aberrations in PMD remain limited. Kamiya et al. reported a progressive increase in coma-like aberrations over an 11-year follow-up period, while spherical aberrations remained relatively stable, highlighting the dominant role of coma in the optical degradation associated with PMD [37]. Similarly, Thibos et al. reported significantly higher levels of coma, spherical aberration, and total RMS aberrations in PMD patients compared with healthy individuals, confirming that PMD is associated with a pronounced increase in HOAs [38]. These aberrations are known to be major determinants of visual quality, particularly under mesopic and scotopic conditions, where pupil dilation exacerbates their optical impact.

Only one study to date has evaluated corneal aberrometry following lamellar crescentic wedge resection in PMD. Genç et al. reported a significant reduction in coma- and spherical-like aberrations over a mean follow-up period of 14 months; however, changes in trefoil aberrations did not reach statistical significance [12]. In contrast to this limited evaluation, our study provides a more comprehensive and long-term analysis of both individual aberration components and total aberration metrics.

In the present study, HOAs were analyzed in two complementary ways:

1. as individual aberration components (coma, spherical aberration, trefoil, and tetrafoil), and
2. as total optical distortion based on RMS values, which represent the cumulative effect of these individual aberrations.

This dual approach allowed us to determine not only whether total aberrations improved after surgery, but also which specific aberrations were primarily responsible for changes in RMS values.

When comparing preoperative and postoperative measurements within the PMD group, significant improvements were noted in several aberration parameters at 3, 6, 12, and 24 months following surgery. Notably, a significant reduction in 4 mm anterior trefoil aberrations was noted, beginning in the 6th postoperative month, with further improvement observed at longer follow-up intervals. In addition, a significant reduction in 4 mm posterior coma was detected as early as the 3rd postoperative month, suggesting an early biomechanical and optical response to lamellar wedge resection.

Our PMD patients exhibited significantly higher levels of 4 mm anterior coma, trefoil, spherical aberration, and tetrafoil at baseline and at most postoperative time points when compared with the healthy control group. However, by the 24-month follow-up, 4 mm tetrafoil values in PMD patients had approached those of the control group, indicating partial normalization of specific aberration components over time. It is worth noting that 4 mm anterior RMS values did not differ significantly between the PMD patients and controls, suggesting that although individual aberrations remained elevated, their combined effect within the central optical zone was relatively well compensated.

A similar pattern was observed for 4 mm posterior aberrations, where PMD patients demonstrated significantly higher posterior coma, trefoil, and spherical aberrations preoperatively and at several postoperative intervals. Nevertheless, the posterior RMS values remained comparable to those of the control group at all time points, indicating that improvements in dominant aberrations—particularly coma and trefoil—may have mitigated their overall impact on total optical quality.

An analysis of aberrations within the 6 mm optical zone yielded clinically important findings. Significantly higher 6 mm anterior coma and trefoil aberrations were noted in the PMD group both pre- and postoperatively when compared with the healthy controls. These aberrations are especially relevant under mesopic and scotopic conditions, during which pupil dilation exposes the peripheral cornea and amplifies optical distortions, leading to such symptoms as glare, halos, reduced contrast sensitivity, and impaired night vision. Significant differences were also observed in 6 mm anterior spherical aberrations, particularly at the early postoperative time points. Despite these differences in individual aberrations, 6 mm anterior RMS values did not differ significantly between the groups, supporting the suggestion that the total aberration burden was largely driven by specific components rather than a uniform increase across all aberrations.

For 6 mm posterior aberrations, significant differences were detected in posterior coma, spherical aberration, and tetrafoil at multiple postoperative time points, whereas the posterior trefoil values remained comparable to those of the control group. Interestingly, 6 mm posterior RMS values showed a transient reduction at postoperative months 3 and 6, indicating a temporary improvement in total posterior optical quality, which may reflect early corneal remodeling following surgery.

Overall, these findings reveal coma and trefoil aberrations to be the dominant contributors to optical degradation in PMD, and that improvements in total RMS values following lamellar wedge resection are primarily driven by reductions in these specific aberrations rather than uniform changes across all HOA components. Furthermore, the differential behavior of aberrations within the 4 mm and 6 mm zones underscores the importance of evaluating both central and peripheral optical zones, as improvements in central vision may not fully reflect visual performance under real-life conditions, particularly in low-light environments.

Our study is unique in that it represents the first comprehensive, long-term evaluation of corneal topography and both anterior and posterior HOAs in multiple optical zones following lamellar wedge resection in PMD patients. The inclusion of a control group and the extended follow-up period of up to 24 months provide a robust framework for the interpretation of both structural and functional outcomes. Furthermore, our detailed analysis offers valuable insight into the optical mechanisms underlying visual improvement after lamellar wedge resection and may serve as an important reference for future studies of this rare corneal ectatic disorder.

Despite its strengths, this study has some limitations that should be acknowledged. First, the sample size was relatively small, which can be attributed to the rarity of this corneal ectatic disorder, although it should be noted that the number of patients included in our study is comparable to, or even exceeds, that of previously published studies of surgical outcomes in PMD. Moreover, the rarity of the disease inherently limits the feasibility of large-scale or randomized studies, particularly those involving long-term follow-up.

Second, the study was conducted at a single center and lacked randomization. Nevertheless, the homogeneous surgical technique, standardized postoperative follow-up protocol, and consistent use of the same imaging modality allowed for reliable longitudinal comparisons within the PMD cohort and against the healthy control group.

Another limitation is the absence of very early postoperative aberration measurements, although our primary objective was to assess mid- to long-term optical and topographic stability following lamellar wedge resection rather than short-term postoperative fluctuations, which are often influenced by transient corneal edema, wound healing, and suture-related effects.

Finally, corneal aberration measurements are inherently dependent on imaging systems and analysis algorithms. To minimize variability, all measurements were obtained using the same device and protocol throughout the study period, ensuring internal consistency across all time points.

Despite these limitations, our study provides the most comprehensive and longest follow-up evaluation to date of anterior and posterior corneal aberrations in the 4 mm and 6 mm optical zones following lamellar wedge resection in PMD patients. Our detailed analysis of the individual aberration components alongside total RMS values offers novel insights into the optical mechanisms underlying visual improvement after surgery and constitutes a meaningful contribution to the existing literature.

## 5. Conclusion

PMD is a rare and slowly progressive corneal ectatic disorder, and surgical series involving lamellar crescentic wedge resection are particularly limited due to the low prevalence of the disease. Consequently, available evidence is largely restricted to small case series with relatively short follow-up periods. Addressing this gap in the literature, the present study is one of the first investigations to analyze the long-term follow-up of patients following lamellar wedge resection, with a comprehensive evaluation of both anterior and posterior corneal HOAs across different optical zones.

In conclusion, lamellar crescentic wedge resection appears to be an effective and safe surgical option for patients with advanced PMD, providing significant and sustained improvements in visual acuity, corneal astigmatism, and HOAs, while avoiding the risks associated with penetrating procedures, such as graft rejection, glaucoma, and open-eye complications. The findings of this study suggest that careful assessment of both individual aberrations and total RMS in multiple optical zones is essential for the accurate evaluation of visual quality outcomes in PMD. Future prospective studies involving larger patient populations and earlier postoperative assessments may further refine patient selection criteria and optimize surgical outcomes.

## Figures and Tables

**Figure 1a:**
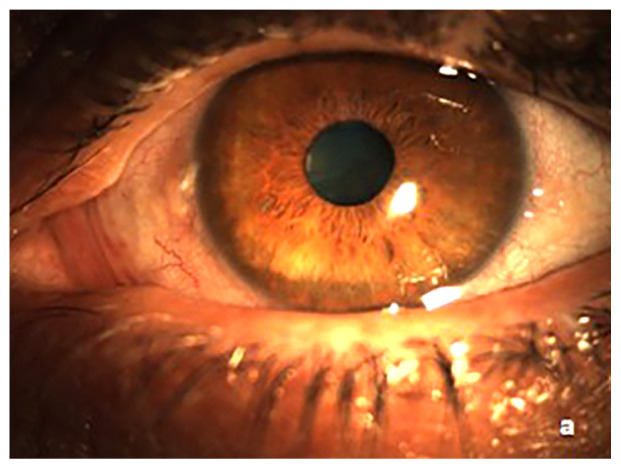
Preoperative image of a PMD patient.

**Figure 1b:**
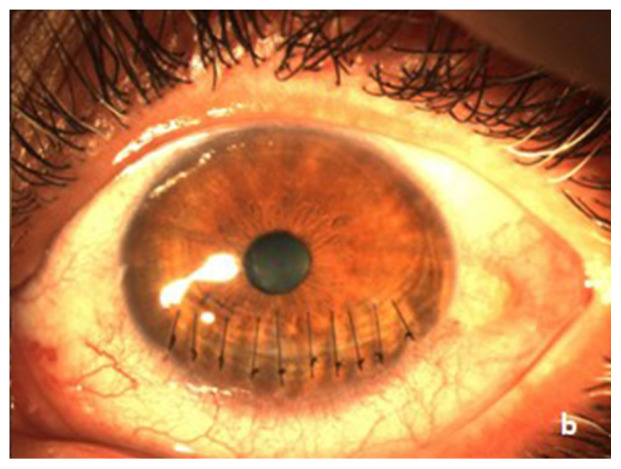
Image of the same patient at postoperative month 3.

**Figure 1c:**
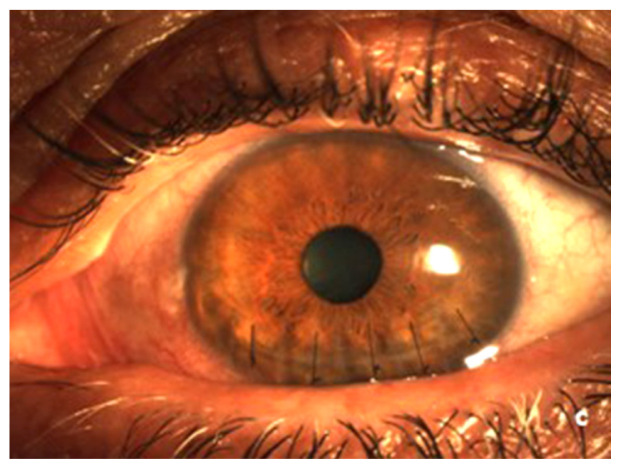
Image of the same patient at postoperative month 6.

**Figure 1d:**
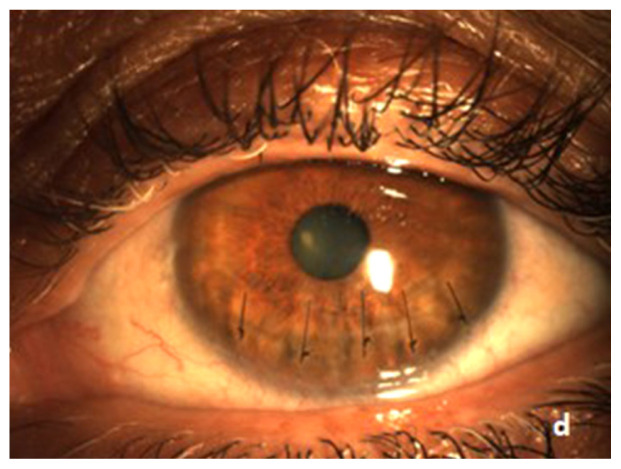
Image of the same patient at postoperative month 12.

**Figure 2a:**
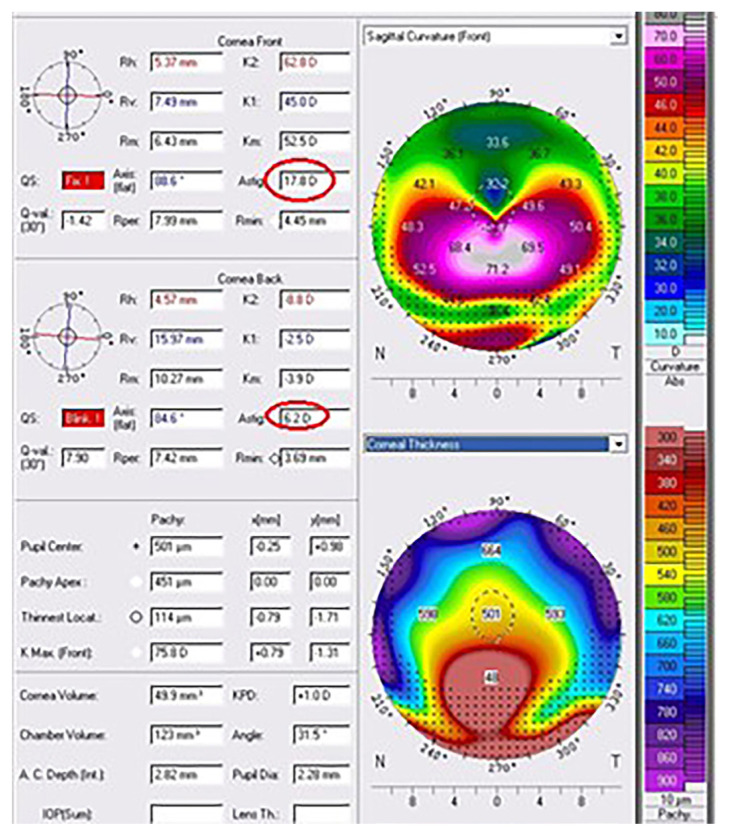
Preoperative Pentacam image of the same patient.

**Figure 2b:**
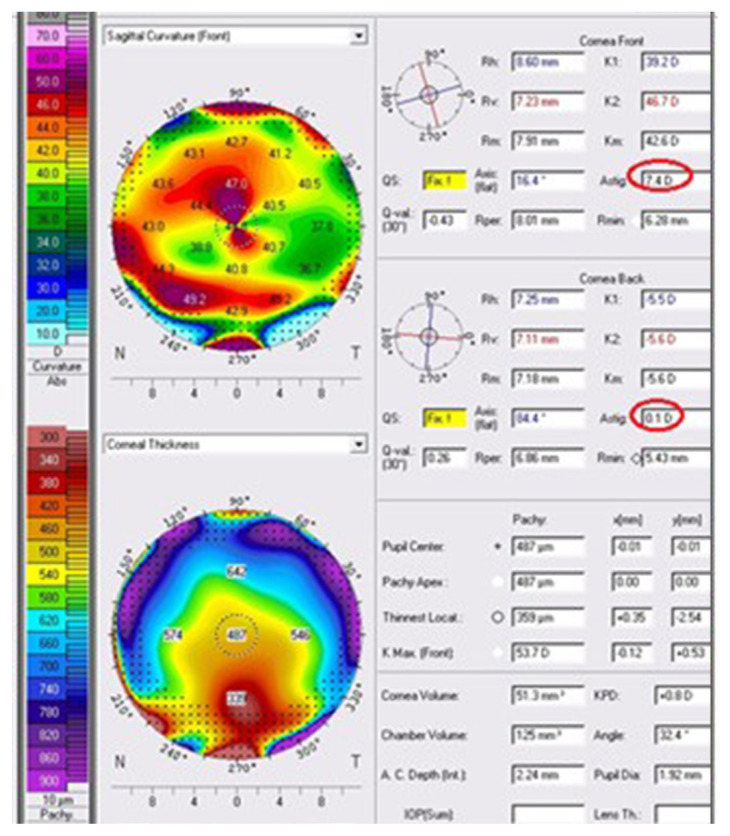
Postoperative Pentacam image of the same patient at month 20.

**Table 1:** Friedman and post hoc tests for repeated measures.

Variable	Mean difference±SD	p-value	Variable	Mean difference±SD	p-value
LogMAR		0.013*	4 mm anterior trefoil		0.022*
Preoperative vs. postoperative month 3	0.682±0.277	0.602	Preoperative vs. postoperative 3-month	−0.070±0.542	0.197
Preoperative vs. postoperative month 6	0.876±0.127	0.014*	Preoperative vs. postoperative 6-month	−0.848±0.646	0.039*
Preoperative vs. postoperative month 12	0.876±0.127	0.014*	Preoperative vs. postoperative 12-month	−0.117±0.180	0.796
Preoperative vs. postoperative month 24	0.651±0.288	0.442	Preoperative vs. postoperative 24-month	0.364±0.254	0.302
Postoperative 3-month vs. postoperative 6-month	0.066±0.121	0.434	Postoperative 3-month vs. postoperative 6-month	−0.382±0.525	0.439
Postoperative 3-month vs. postoperative 12-month	0.162±0.162	0.434	Postoperative 3-month vs. postoperative 12-month	0.253±0.326	0.302
Postoperative 3-month vs. postoperative 24-month	0.046±0.388	1.000	Postoperative 3-month vs. postoperative 24-month	0.416±0.449	0.020*
Postoperative 6-month vs. postoperative 12-month	0.096±0.164	0.823	Postoperative 6-month vs. postoperative 12-month	0.636±0.460	0.071
Postoperative 6-month vs. postoperative 24-month	−0.020±0.411	0.576	Postoperative 6-month vs. postoperative 24-month	0.954±0.755	0.002*
Postoperative 12-month vs. postoperative 24-month	−0.116±0.347	0.576	Postoperative 12-month vs. postoperative 24-month	0.316±0.343	0.197
4 mm posterior coma		0.042*	4 mm posterior coma		0.042*
Preoperative vs. postoperative month 3	1.817±2.267	0.027*	Postoperative 3-month vs. postoperative 12-month	−0.637±0.916	0.683
Preoperative vs. postoperative month 6	2.333±1.905	0.683	Postoperative 3-month vs. postoperative 24-month	−1.255±0.872	0.086
Preoperative vs. postoperative month 12	1.521±1.769	1.000	Postoperative 6-month vs. postoperative 12-month	−0.405±0.717	1.000
Preoperative vs. postoperative month 24	1.34±1.635	1.000	Postoperative 6-month vs. postoperative 24-month	−0.763±1.034	0.237
Postoperative 3-month vs. postoperative 6-month	−0.233±0.531	1.000	Postoperative 12-month vs. postoperative 24-month	−0.359±1.024	1.000

* p < 0.05 (p-values adjusted for multiple comparisons using the Bonferroni correction).

**Table 2:** LogMAR, astigmatism, axis, Kmax.

	Mean±SD						p-value				
	Preoperative	Postoperative month 3	Postoperative month 6	Postoperative month 12	Postoperative month 24	Control	Control vs. preoperative	Control vs. postoperative month 3	Control vs. postoperative month 6	Control vs. postoperative month 12	Control vs. postoperative month 24
LogMAR	1.32±0.36	0.61±0.23	0.53±0.27	0.46±0.33	0.50±0.42	0.02±0.03	0.001**	0.000***	0.001**	0.013*	0.004**
Astigmatism	12.84±6.38	11.52±4.79	8.30±5.43	8.34±4.53	10.18±3.23	7.43±3.93	0.000***	0.001**	0.004**	0.006*	0.004**
Axis	72.60±52.96	63.33±80.96	43.53±69.74	79.70±64.06	95.83±83.54	94.09±9.12	0.336	0.414	0.214	0.524	1.000
Kmax	70.43±11.42	59.90±9.89	55.65±5.02	55.00±3.83	61.25±6.78	49.87±3.66	0.000***	0.001**	0.004**	0.002**	0.004**

* p < 0.05,

** p < 0.005,

*** p < 0.001

**Table 3:** Anterior and posterior 4 mm HOAs.

	Mean±SD						p-value				
Anterior and posterior 4 mm HOAs	Preoperative	Postoperative month 3	Postoperativemonth 6	Postoperative month 12	Postoperative month 24	Control	Control vs. Preoperative	Control vs. Postoperative month 3	Control vs. Postoperative month 6	Control vs. Postoperative month 12	Control vs. Postoperative month 24
Anterior 4 mm coma	3.04±2.39	1.21±085	1.22±1.49	0.84±0.23	1.46±0.88	0.19±.15	0.001**	0.008*	0.016*	0.003**	0.002**
Anterior 4 mm trefoil	1.59±1.68	0.92±0.46	0.83±0.23	1.26±1.53	0.75±0.38	0.13±0.13	0.001**	0.001**	0.004**	0.006*	0.003**
Anterior 4 mm spheric	0.51±0.20	0.34±0.07	0.57±0.54	0.28±0.15	0.50±0.21	0.07±.07	0.001**	0.001**	0.004**	0.019*	0.002**
Anterior 4 mm tetrafoil	0.59±0.34	0.78±0.46	1.20±0.63	0.61±0.20	0.25±0.14	0.14±0.08	0.014*	0.013*	0.004**	0.002*	0.171
Anterior 4 mm RMS	−0.73±3.09	−0.53±2.42	0.18±1.34	1.27±2.49	0.79±1.69	0.02±0.46	0.694	0.755	0.808	0.171	0.354
Posterior 4 mm coma	3.70±2.97	1.35±089	1.19±0.80	2.94±3.10	2.34±1.37	0.59±0.60	0.002**	0.181	0.154	0.019*	0.019*
Posterior 4 mm trefoil	4.22±4.19	2.18±2.00	1.64±0.87	2.89±4.34	1.73±0.58	1.28±1.25	0.072*	0.414	0.461	0.833	0.093
Posterior 4 mm spheric	1.29±0.92	0.99±0.89	0.62±0.61	0.52±0.28	0.68±0.46	0.30±0.14	0.006*	0.059	0.368	0.127	0.222
Posterior 4mm tetrafoil	1.31±1.45	1.08±0.21	1.12±0.55	1.30±0.57	1.25±0.32	0.53±0.25	0.397	0.001**	0.154	0.006*	0.006*
Posterior 4 mm RMS	−0.12±4.17	−1.28±4.38	0.38±1.70	3.42±9.46	−1.67±4.51	−0.05±1.20	0.779	1.000	0.683	0.943	0.833

* p < 0.05,

** p < 0.005

**Table 4:** Anterior and posterior 6 mm HOAs.

	Mean±SD						p-value				
Anterior and posterior 6mm HOA	Preoperative	Postoperative month 3	Postoperative month 6	Postoperative month 12	Postoperative month 24	Control	Control vs. preoperative	Control vs. postoperative month 3	Control vs. postoperative month 6	Control vs. postoperative month 12	Control vs. postoperative month 24
Anterior 6 mm coma	6.84±4.68	1.87±1.38	1.51±1.80	1.58±0.66	2.81±1.79	0.49±0.24	0.001**	0.008*	0.214	0.003**	0.002**
Anterior 6 mm trefoil	2.46±2.43	1.08±0.79	0.74±.35	1.86±3.61	0.73± 0.48	0.28±0.11	0.001**	0.005*	0.028*	0.833	0.030*
Anterior 6 mm spheric	0.85±0.43	0.65± .32	0.62± 0.35	0.60±0.37	0.95±0.58	0.26± 0.23	0.008*	0.043*	0.073	0.065	0.006*
Anterior 6 mm tetrafoil	1.30±.70	1.87±1.06	2.32±0.83	1.43±0.64	0.78±0.31	0.39±0.16	0.020*	0.020*	0.004**	0.002**	0.045*
Anterior 6 mm RMS	−4.3±4.37	−1.51±2.45	−0.26±.49	2.32±5.60	0.31±2.84	0.18± 1.66	0.081	0.181	0.214	0.622	0.943
Posterior 6 mm coma	6.38±5.51	2.42±1.41	2.14±2.12	W4.49±4.09	3.19±2.07	0.93±0.84	0.008*	0.043*	0.283	0.011*	0.065
Posterior 6 mm trefoil	7.23±7.31	3.38±2.17	2.01±1.01	4.22±7.21	2.21±1.63	1.74±1.51	0.059	0.108	0.368	1.000	0.354
Posterior 6 mm spheric	2.76±1.43	2.19±1.91	1.42±1.22	1.24± 0.96	1.30±0.89	0.55±0.22	0.001**	0.029*	0.048*	0.127	0.019*
Posterior 6 mm tetrafoil	3.40±3.40	2.50±0.98	3.11±0.55	3.15±1.68	2.86±1.08	1.04±0.46	0.181	0.003**	0.004**	0.019*	0.003**
Posterior 6 mm RMS	−2.01±3.84	−3.33±6.94	−0.90±2.05	3.92± 15.41	−3.58± 7.32	1.73±2.04	0.181	0.043*	0.028*	0.127	0.284

* p < 0.05,

** p < 0.005.

## References

[1] Jhanji V Ahmad S Amescua G Cheung AY Choi DS American Academy of Ophthalmology Preferred Practice Pattern Cornea/External Disease Panel Corneal Ectasia Preferred Practice Pattern® Ophthalmology 2024 131 4 205 246 10.1016/j.ophtha.2023.12.038

[2] Sahu J Raizada K Pellucid marginal corneal degeneration 2023 Aug 28 Stat Pearls [Internet] Treasure Island (FL) StatPearls Publishing 2024 Book 32965985

[3] Mosca L Carlà MM Guccione L Vico U Scartozzi L Topographical and functional analysis of different surgical strategies for advanced pellucid marginal degeneration: a long term follow-up European Journal of Ophthalmology 2025 35 3 884 892 10.1177/11206721241297324 39533956

[4] Omar Yousif M Elkitkat RS Hamza MN Abdelsadek Alaarag N Application of a revised tissue saving protocol for combined topography-guided photorefractive keratectomy and cross-linking in a cohort having pellucid marginal degeneration Clinical Ophthalmology 2024 18 303 311 10.2147/OPTH.S449766 38317793 PMC10840534

[5] Irajpour M Noorshargh P Peyman A Corneal cross-linking in pellucid marginal degeneration: evaluation after five years Journal of Current Ophthalmology 2022 34 2 229 233 10.4103/joco.joco_16_22 36147277 PMC9487015

[6] Cagil N Sarac O Yesilirmak N Caglayan M Uysal BS Transepithelial phototherapeutic keratectomy followed by corneal collagen crosslinking for the treatment of pellucid marginal degeneration: long-term results Cornea 2019 38 8 980 985 10.1097/ICO.0000000000002003 31107284

[7] Barbara A Shehadeh-Masha’our R Zvi F Garzozi HJ Management of pellucid marginal degeneration with intracorneal ring segments Journal of Refractive Surgery 2005 21 3 296 298 10.3928/1081-597X-20050501-15 15977889

[8] MacLean H Robinson LP Wechsler AW Long-term results of corneal wedge excision for pellucid marginal degeneration Eye (London) 1997 11 Pt 5 613 617 10.1038/eye.1997.164 9474305

[9] Millar MJ Maloof A Deep lamellar keratoplasty for pellucid marginal degeneration: review of management options for corneal perforation Cornea 2008 27 8 953 956 10.1097/ICO.0b013e31816ed516 18724162

[10] Maccherön LJ Daya SM Wedge resection and lamellar dissection for pellucid marginal degeneration Cornea 2012 31 6 708 715 10.1097/ICO.0b013e31824000e3 22575848

[11] Erdinest N London N Landau D Barbara R Barbara A Higher order aberrations in keratoconus International Ophthalmology 2024 10 44 1 172 10.1007/s10792-024-03118-5 38594548

[12] Genç S Çakir H Güler E Çalli Ü Refractive and corneal aberrometric changes after crescentic lamellar wedge resection in pellucid marginal degeneration Eye Contact Lens 2018 44 Suppl 2 S76 S80 10.1097/ICL.0000000000000409 28737665

[13] Oie Y Maeda N Kosaki R Suzaki A Hirohara Y Characteristics of ocular higher-order aberrations in patients with pellucid marginal corneal degeneration Journal of Cataract and Refractive Surgery 2008 34 11 1928 1934 10.1016/j.jcrs.2008.06.038 19006740

[14] Mounir A Abdellah MM Awny I Aldghaimy AH Mostafa EM Demographic, clinical and tomographic characteristics of pellucid marginal degeneration patients in South Egyptian population International Ophthalmology 2022 42 10 3237 3242 10.1007/s10792-022-02326-1 36001208 PMC9509300

[15] Moshirfar M Edmonds JN Behunin NL Christiansen SM Current options in the management of pellucid marginal degeneration Journal of Refractive Surgery 2014 30 7 474 485 10.3928/1081597X-20140429-02 24816208

[16] Gonçalves TB Nosé AFB Pereira NC Forseto ADS Partial-thickness intrastromal lamellar keratoplasty for corneal pellucid marginal degeneration Cornea 2021 40 12 1620 1623 10.1097/ICO.0000000000002671 34749384

[17] Kanellopoulos AJ Combined photorefractive keratectomy and corneal cross-linking for keratoconus and ectasia: The Athens Protocol Cornea 2023 42 10 1199 1205 10.1097/ICO.0000000000003320 37669421 PMC10476591

[18] Yu AC Spena R Pellegrini M Bovone C Busin M Deep anterior lamellar keratoplasty: current status and future directions Cornea 2022 41 5 539 544 10.1097/ICO.0000000000002840 34759197

[19] Feizi S Javadi MA Karimian F Bayat K Bineshfar N Penetrating keratoplasty versus deep anterior lamellar keratoplasty for advanced stage of keratoconus American Journal of Ophthalmology 2023 248 107 115 10.1016/j.ajo.2022.11.019 36476362

[20] Aldebasi T Gangadharan S Alshammari YS Alruhaimi SS Alrashid SO Comparison of clinical outcomes, complications and patient satisfaction following deep anterior lamellar keratoplasty and penetrating keratoplasty BMC Ophthalmology 2024 15 24 1 501 10.1186/s12886-024-03766-2 39548416 PMC11566242

[21] Santodomingo-Rubido J Carracedo G Suzaki A Villa-Collar C Vincent SJ Keratoconus: an updated review Contact Lens and Anterior Eye 2022 45 3 101559 10.1016/j.clae.2021.101559 34991971

[22] Niazi S Doroodgar F Hashemi Nazari S Rahimi Y Alió Del Barrio JL Refractive surgical approaches to keratoconus: A systematic review and network meta-analysis Survey of Ophthalmology 2024 69 5 779 788 10.1016/j.survophthal.2024.04.008 38710236

[23] Deshmukh R Ong ZZ Rampat R Alió Del Barrio JL Barua A Management of keratoconus: an updated review Frontiers in Medicine (Lausanne) 2023 10 1212314 10.3389/fmed.2023.1212314 PMC10318194 37409272

[24] Kymionis GD Karavitaki AE Kounis GA Portaliou DM Yoo SH Management of pellucid marginal corneal degeneration with simultaneous customized photorefractive keratectomy and collagen crosslinking Journal of Cataract and Refractive Surgery 2009 35 7 1298 1301 10.1016/j.jcrs.2009.03.025 19545822

[25] Wileman JM Price MO Price FW Jr Case of progressive keratoconus with newly diagnosed pellucid marginal degeneration after corneal cross-linking Cornea 2024 43 2 257 260 10.1097/ICO.0000000000003397 37733982

[26] Borgardts K Menzel-Severing J Geerling G Seiler TG Indikationsstellung zum Crosslinking und klinische Ergebnisse neuer kornealer Crosslinking-Techniken Treatment indications for corneal crosslinking and clinical results of new corneal crosslinking techniques Ophthalmologe 2022 119 4 350 357 (in German). 10.1007/s00347-022-01579-6 35147774

[27] Spadea L Maraone G Cagini C Sliding keratoplasty followed by transepithelial iontophoresis collagen cross-linking for pellucid marginal degeneration Journal of Refractive Surgery 2016 32 1 47 50 10.3928/1081597X-20151119-02 26812714

[28] Durán JA Rodriguez-Ares MT Torres D Crescentic resection for the treatment of pellucid corneal marginal degeneration Ophthalmic Surgery 1991 22 3 153 156 10.3928/1542-8877-19910301-08 2030897

[29] Varley GA Macsai MS Krachmer JH The results of penetrating keratoplasty for pellucid marginal corneal degeneration American Journal of Ophthalmology 1990 110 2 149 152 10.1016/S0002-9394(14)76983-1 2378379

[30] Michael T Ioannis G Ferdinardo M Ioannis A Endothelium sparing - air-assisted wedge resection for the treatment of pellucid marginal degeneration Indian Journal of Ophthalmology 2024 72 Suppl 2 S314 S318 10.4103/IJO.IJO_3033_22 38146974 PMC11624646

[31] Cameron JA Results of lamellar crescentic resection for pellucid marginal corneal degeneration American Journal of Ophthalmology 1992 113 3 296 302 10.1016/S0002-9394(14)71582-X 1543223

[32] García de Oteyza G Borasio E Ruíz-Santos M Julio G Barraquer RI Analysis of visual and refractive results after wedge resection for high astigmatism after penetrating keratoplasty in keratoconus European Journal of Ophthalmology 2022 11206721221144656 10.1177/11206721221144656 36537167

[33] AlSaadi A AlMaazmi A A sequential approach to the management of an advanced case of pellucid marginal degeneration American Journal of Ophthalmology Case Reports 2023 32 101966 10.1016/j.ajoc.2023.101966 38077780 PMC10698564

[34] Busin M Kerdraon Y Scorcia V Zambianchi L Matteoni S Combined wedge resection and beveled penetrating relaxing incisions for the treatment of pellucid marginal corneal degeneration Cornea 2008 27 5 595 600 10.1097/ICO.0b013e318166c40c 18520511

[35] Maccheron LJ Daya SM Wedge resection and lamellar dissection for pellucid marginal degeneration Cornea 2012 31 6 708 715 10.1097/ICO.0b013e31824000e3 22575848

[36] Kymionis G Voulgari N Samutelela E Kontadakis G Tabibian D Combined corneal wedge resection and corneal cross-linking for pellucid marginal degeneration: a first report Therapeutics and Clinical Risk Management 2019 15 1319 1324 10.2147/TCRM.S210606 31814727 PMC6858838

[37] Kamiya K Hirohara Y Mihashi T Progression of pellucid marginal degeneration and higher-order wavefront aberration of the cornea Japanese Journal of Ophthalmology 2003 47 5 523 525 10.1016/S0021-5155(03)00126-6 12967872

[38] Thibos LN Hong X Clinical applications of the Shack-Hartmann aberrometer Optometry and Vision Science 1999 76 12 817 825 10.1097/00006324-199912000-00016 10612402

